# Nitrogen Removal Characteristics of a Newly Isolated Indigenous Aerobic Denitrifier from Oligotrophic Drinking Water Reservoir, *Zoogloea* sp. N299

**DOI:** 10.3390/ijms160510038

**Published:** 2015-05-04

**Authors:** Ting-Lin Huang, Shi-Lei Zhou, Hai-Han Zhang, Shi-Yuan Bai, Xiu-Xiu He, Xiao Yang

**Affiliations:** School of Environmental and Municipal Engineering, Xi’an University of Architecture and Technology, Xi’an 710055, China; E-Mails: ZSLZhouShilei@126.com (S.-L.Z.); zhanghaihan@xauat.edu.cn (H.-H.Z.); yuanired0502@163.com (S.-Y.B.); hexiuxiuxauat@126.com (X.-X.H.); YangXiaoxauat@126.com (X.Y.)

**Keywords:** aerobic denitrification, *Zoogloea* sp. N299, 16S rRNA, *nap*A gene, reservoir water

## Abstract

Nitrogen is considered to be one of the most widespread pollutants leading to eutrophication of freshwater ecosystems, especially in drinking water reservoirs. In this study, an oligotrophic aerobic denitrifier was isolated from drinking water reservoir sediment. Nitrogen removal performance was explored. The strain was identified by 16S rRNA gene sequence analysis as *Zoogloea* sp. N299. This species exhibits a periplasmic nitrate reductase gene (*nap*A). Its specific growth rate was 0.22 h^−1^. Obvious denitrification and perfect nitrogen removal performances occurred when cultured in nitrate and nitrite mediums, at rates of 75.53% ± 1.69% and 58.65% ± 0.61%, respectively. The ammonia removal rate reached 44.12% ± 1.61% in ammonia medium. *Zoogloea* sp. N299 was inoculated into sterilized and unsterilized reservoir source waters with a dissolved oxygen level of 5–9 mg/L, pH 8–9, and C/N 1.14:1. The total nitrogen removal rate reached 46.41% ± 3.17% (sterilized) and 44.88% ± 4.31% (unsterilized). The cell optical density suggested the strain could survive in oligotrophic drinking water reservoir water conditions and perform nitrogen removal. Sodium acetate was the most favorable carbon source for nitrogen removal by strain N299 (*p* < 0.05). High C/N was beneficial for nitrate reduction (*p* < 0.05). The nitrate removal efficiencies showed no significant differences among the tested inoculums dosage (*p* > 0.05). Furthermore, strain N299 could efficiently remove nitrate at neutral and slightly alkaline and low temperature conditions. These results, therefore, demonstrate that *Zoogloea* sp. N299 has high removal characteristics, and can be used as a nitrogen removal microbial inoculum with simultaneous aerobic nitrification and denitrification in a micro-polluted reservoir water ecosystem.

## 1. Introduction

During the past few decades, more and more nitrogen has been discarded into the fresh water ecosystem, leading to serious environmental problems [1,2,3,4], such as eutrophication, algae bloom, and unsafe water [5,6], especially in drinking water reservoirs [7,8]. Nitrogen removal in freshwater ecosystems is important. Physical (air stripping) [9] and chemical techniques (chemical precipitation) [10] are widely used to remove nitrogen from wastewater, as the traditional biological method (nitrification by autotrophs under aerobic conditions and denitrification by heterotrophs under anaerobic conditions) is impractical [11]. Conventional biological denitrification only occurs under anaerobic or anoxic conditions with the reduction from nitrate to nitrogen gas [12]. Oxygen inhibits the reaction steps, which makes them impractical in natural waters, especially reservoirs [13].

Robertson and Kuenen’s discovery of aerobic denitrification bacteria *Thiosphaera pantotropha* [14] at a denitrifying, sulfide-oxidizing wastewater treatment plant, demonstrated a novel method of nitrogen removal, which is not limited to oxygen [14,15,16]. Microbiologists have defined aerobic denitrification as the co-respiration or co-metabolism of oxygen and nitrate [17]. Aerobic denitrification has attractive advantages compared to conventional anaerobic denitrification [18,19]: nitrification and denitrification can occur in the same system [20], and denitrification can cause sufficient alkalinity to balance the acidity of nitrification [21]. There are recent reports of aerobic denitrification bacteria isolated from canals [22], ponds [23], and soils [24]; the dominant species include *Thiosphaera pantotropha* [25], *Alcaligenes faecalis* [12,26], *Citrobacter diversus* [27], *Pseudomonas stutzeri* [28], and *Rhodococcus* sp. [29].

Compared with strains isolated in massive amounts from other environmental systems [21,22,23], aerobic denitrifiers are rarely isolated from reservoirs, and there is limited research on using them to bioremediate reservoir ecosystems [30,31]. Several studies have illustrated the difficulties of removing nitrogen from source water, such as, low carbon source levels are not conducive to the growth of heterotrophic aerobic denitrifiers; cause acclimation problems [31]; and limit of the denitrification process in freshwater reservoirs [32,33]. Due to its low concentration as a pollutant in natural water system [12,33,34,35]. Therefore, limited studies were focused on aerobic denitrifiers’ characteristics at removing nitrogen from oligotrophic drinking water reservoirs. Our research group has reported the water quality of aerobic denitrifiers elsewhere [36,37,38,39,40,41], and demonstrated *Rhizobium* sp. PY8 [38] had a good ability of simultaneous nitrification and denitrification under conditions of initial pH 6.0–10.0, temperature 25–30 °C, C/N ratio 1.0–9.0 conditions. The low temperature resistant and oligotrophic denitrifying functional microorganism groups could be constructed by self-adjustment and eco-recombination and the results showed that the maximum removal rate of nitrate and TN could reach 46% and 53%, respectively, during operation under the conditions of temperature about 10 to 18 °C of the source water quality [39]. We carried out pilot research on micro-pollutant removal in the raw water by a combined process of water-lifting aeration and oligotrophic biofilm [37], and the result showed that the nitrogen removal effects can meet the requirements of class III based on the Chinese Surface Water Environment Quality Standard (GB3838-2002).

To this end, we isolated 196 strains using enrichment and screening processes. We found that the N299 strain has perfect performance on nitrogen removal in aerobic conditions with low pollutant concentration. Therefore, the objectives of the present work were to determine the taxonomic status using the 16S rRNA method, to examine the *nap*A gene, to determine nitrogen removal performance in nutrient medium and micro-polluted raw water, and to explore the effect of different factors on nitrate removal. The results can be useful for using aerobic denitrifiers for micro-pollution reservoir bioremediation.

## 2. Results

### 2.1. Identification and Phylogenetic Analysis of Aerobic Denitrifier N299

In the preliminary experiment with enrichment culture isolation, 196 strains were isolated. The isolated strain N299 with high nitrogen removal efficiency was obtained in this study, and was stored on SM (screening medium) slant medium at 4 °C and on SM Glycerin medium at −20 °C.

**Figure 1:**
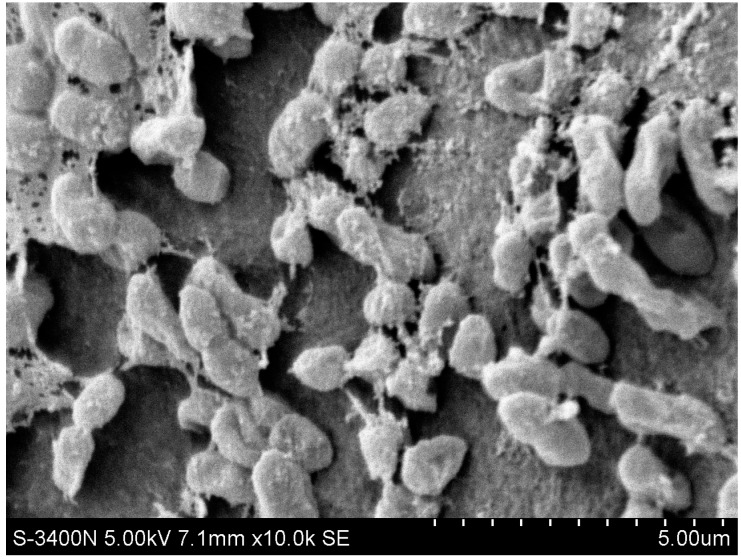
Scanning Electron Microscope (SEM) image of N299 strain.

**Figure 2:**
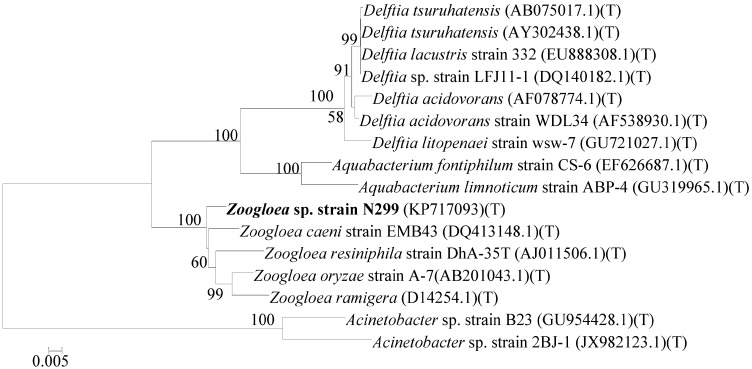
Phylogenetic tree based on the comparison of partial 16S rRNA gene sequences of the N299 strain and other culture strain sequences. The genetic tree was constructed using a neighbor-joining method with Bootstrap values of 1000 replications.

**Figure 3:**
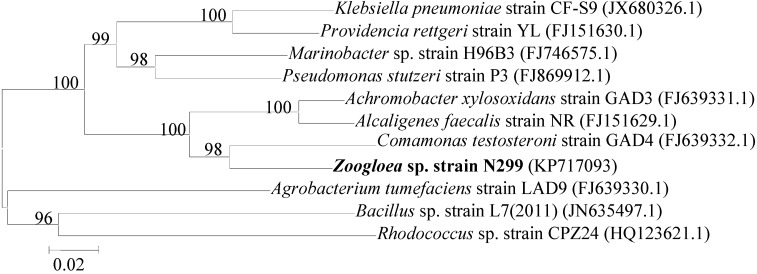
Phylogenetic tree based on the 16S rRNA gene sequence of N299 strain and other previously studied aerobic denitrifiers. The genetic tree was constructed using a neighbor-joining method with Bootstrap values of 1000 replications.

The N299 strain was a gram-negative, aerobic, rod-shaped bacterium, and its size was approximately (0.5–1.0) × (1.5–3.0) μm (Figure 1). The 1461 bp of 16S rRNA sequences of N299 was obtained and exhibited 97.85% similarity with *Zoogloea caeni* strain EMB43 (Figure 2). A neighbor-joining phylogenetic tree was constructed showing the phylogenetic relationships between strain N299 and other culture strains (Figure 2). This revealed the strain N299 and *Zoogloea caeni* strain EMB43 (type culture strain) were in the same group. Therefore, the N299 strain was identified as *Zoogloea*. A neighbor-joining phylogenetic tree showing the phylogenetic relationships between strain N299 and other previously studied aerobic denitrifiers was also constructed (Figure 3). Results showed that *Zoogloea* sp. N299 and *Comamonas testosteroni* strain GAD4 (aerobic denitrifier) were in the same group. Therefore, the strain was affiliated with *Zoogloea* sp. N299. The nucleotide sequence of N299 was submitted into GenBank nucleotide sequence databases under the accession number KP717093, and the strain was also deposited to the China General Microbiological Culture Collection Center (CGMCC) under the number 4.7825.

### 2.2. napA Examination

**Figure 4:**
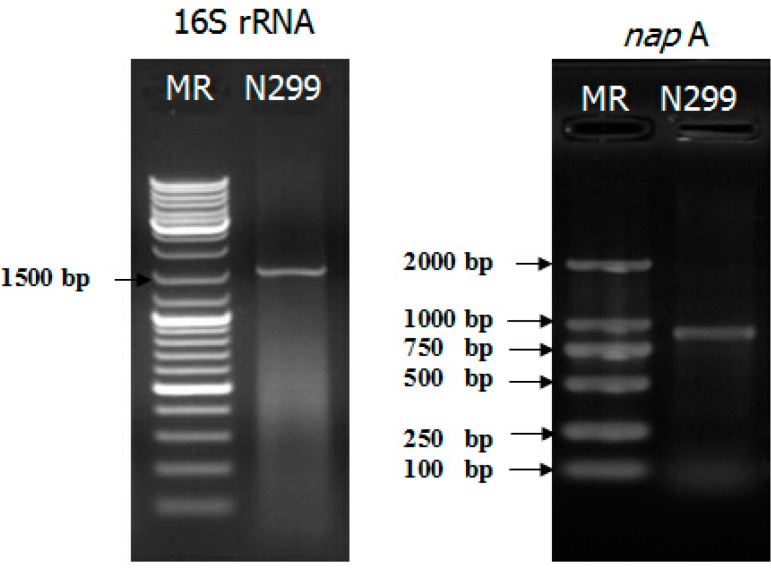
Polymerase Chain Reaction (PCR) products of 16S rRNA and *nap*A gene of N299 strain.

The nitrate reductase gene is widely used as functional marker to identify the aerobic denitrifying bacteria. To examine whether the N299 strain was an aerobic denitrifier, the genes encoding periplasmic nitrate reductase were amplified, and an 877 bp fragment *nap*A was obtained (Figure 4).

### 2.3. Growth Characteristics of N299

**Figure 5:**
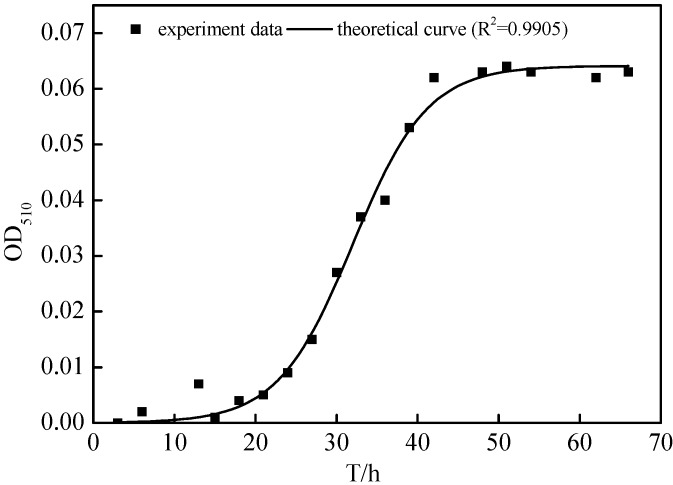
Growth curve of N299 strain in liquid SM (screening medium) medium at pH 7.0–7.5: CH_3_COONa (0.1 g/L); NaNO_3_ (0.02 g/L); K_2_HPO_4_·3H_2_O (0.02 g/L); CaCl_2_ (0.01 g/L); MgCl_2_·6H_2_O (0.01 g/L).

Figure 5 shows the growth curve of the N299 strain as a sigmoid curve. The first 18 h comprised the lag phase, followed by a 16 h logarithmic growth phase. The last 34 h was the stationary phase. During the growth period, the OD_510_ (optical density) of the strain N299 increased from 0.004 to 0.062. *a* = 0.064, *c* = 0.004, and μ = 0.22 h^−1^, with a correlation coefficient of 0.9905. The generation time for N299 was 3.15 h.

### 2.4. Denitrification of Zoogloea sp. N299 Using Nitrate and Nitrite as Sole Nitrogen Source

**Figure 6:**
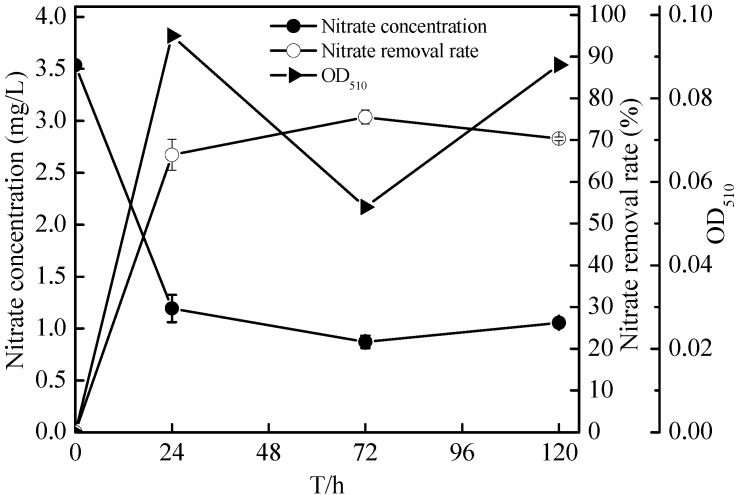
Changes in nitrate, nitrate removal rates, and OD_510_ of N299 strain growth in nitrate nitrogen medium.

**Figure 7:**
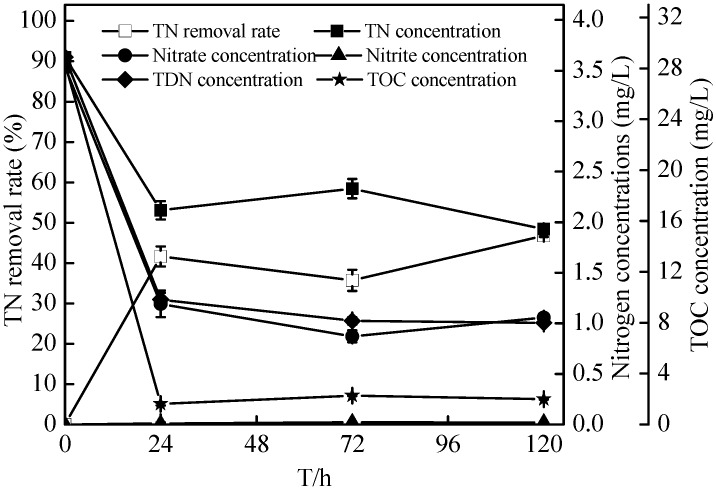
Changes in total nitrogen, total dissolved nitrogen, nitrate, nitrite, and total organic carbon (TOC) concentrations in nitrate nitrogen medium.

Under aerobic conditions (dissolved oxygen, DO = 7.0–8.0 mg/L), *Zoogloea* sp*.* N299 demonstrated obviously denitrification performance. As shown in Figure 6 and Figure 7, in 72 h, nitrate decreased from 3.54 ± 0.03 to 0.87 ± 0.06 mg/L, and nitrite increased from 0 to 0.02 ± 0.00 mg/L (no nitrite accumulation). Meanwhile, the total nitrogen (TN) decreased from 3.63 ± 0.03 to 1.93 ± 0.01 mg/L and total dissolved nitrogen (TDN) decreased from 3.63 ± 0.03 to 1.00 ± 0.02 mg/L in 120 h. The nitrogen removal effects can meet the requirements of class III of Chinese Surface Water Environment Quality Standard (GB3838-2002). In 120 h, the removal rates of TN and TDN reached 46.79% ± 0.30% and 72.30% ± 0.52%, respectively. As seen in Figure 7, the nitrate decreased from 3.54 ± 0.03 to 1.19 ± 0.13 mg/L, and the total organic carbon (TOC) from 28.38 ± 0.69 to 1.62 ± 0.13 mg/L in 72 h. Meanwhile, the OD_510_ of the N299 strain increased from 0 to 0.095, which was lag phase. The N299 strain resulted in simultaneous organic matter and nitrate removal. It was suggested that utilization of organic matter and degradation of nitrate nitrogen took place simultaneously in a true heterotrophic process in logarithmic growth phase. The denitrification of N299 became weak at low C/N (0.97/1) in 72 h, and the denitrification stopped. Because carbon is essential for cell growth and nitrate reduction processes, the optimal quantity of carbon is a key parameter in the denitrification process.

**Figure 8:**
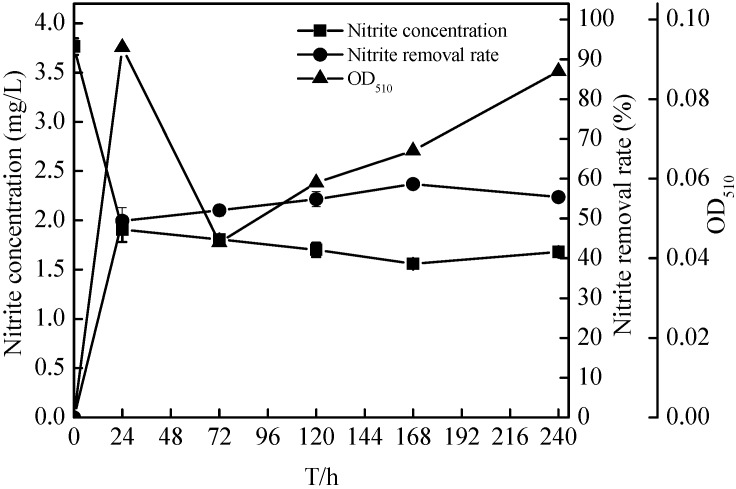
Changes in nitrite, nitrite removal rates, and OD_510_ of strain N299 growth in nitrite nitrogen medium.

**Figure 9:**
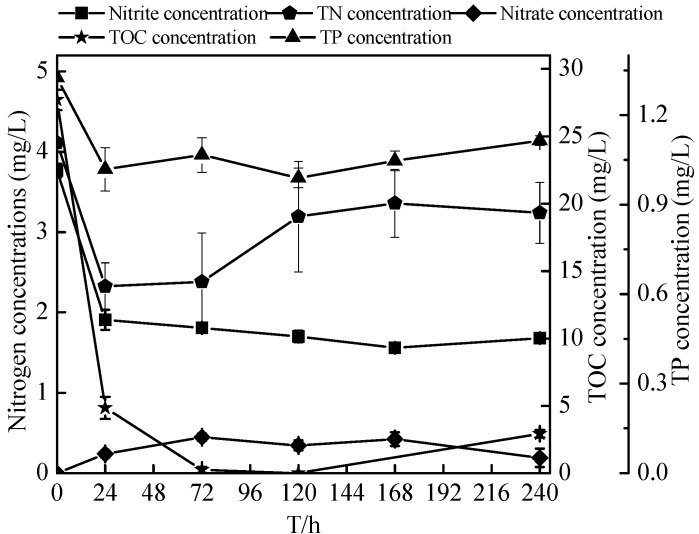
Changes in TN (total nitrogen), nitrate, nitrite, TP (total phosphorus), and TOC (total organic carbon) concentrations in nitrite nitrogen medium.

Few aerobic denitrifiers using nitrite as a sole nitrogen source were identified. Considering the nitrogen level of oligotrophic reservoir and inflow rivers, therefore, the initial nitrite was fixed 3–4 mg/L, and the denitrification activity of N299 strain using nitrite as sole nitrogen was assessed. Figure 8 and Figure 9 showed the time courses of the concentration, TN, nitrite, nitrate, TP (total phosphorus), OD_510_ and TOC levels at initial 3.76 mg/L nitrite. The removal of nitrite and TOC correlated strongly with the growth rate of isolate N299, as shown in Figure 8 and Figure 9, with the fastest removal rates occurring during the log phase (first 24 h). The nitrite decreased from 3.76 ± 0.08 to 1.56 ± 0.01 mg/L, and TOC decreased from 27.70 ± 0.75 to 0 mg/L in 120 h. The C/N (TOC/TN) ranged from 6.74/1 to 2.08/1 in 24 h, 0.10/1 in 72 h, 0.00/1 in 120 h, to 0.91/1 in 240 h, respectively. Because of the low C/N, the denitrification of N299 became weak. Meanwhile, with the strain’s growth, the TN and TP removal rates reached 21.38% ± 9.22% and 15.97% ± 1.25%. At the end of the experiment, only the nitrate increased (to 0.19 ± 0.11 mg/L). The N299 demonstrated denitrification by utilizing the nitrite as the sole nitrogen source.

### 2.5. Nitrification Characteristics of the Zoogloea sp. N299

**Figure 10:**
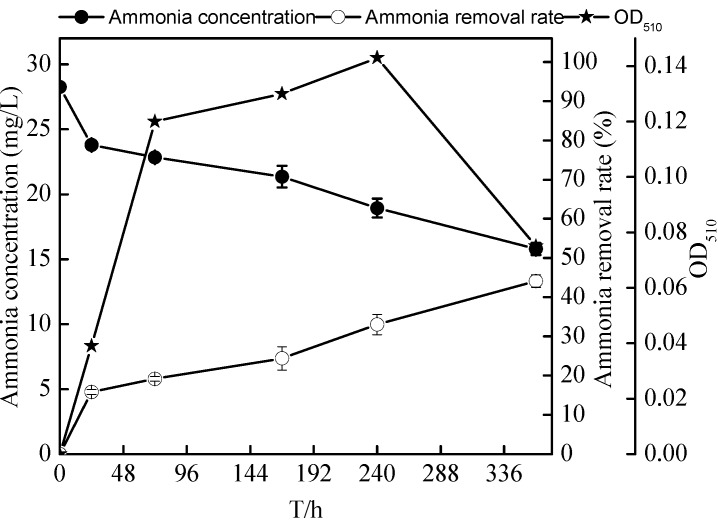
Changes in ammonia, ammonia removal rates, and OD_510_ of strain N299 growth in ammonia nitrogen medium.

**Figure 11:**
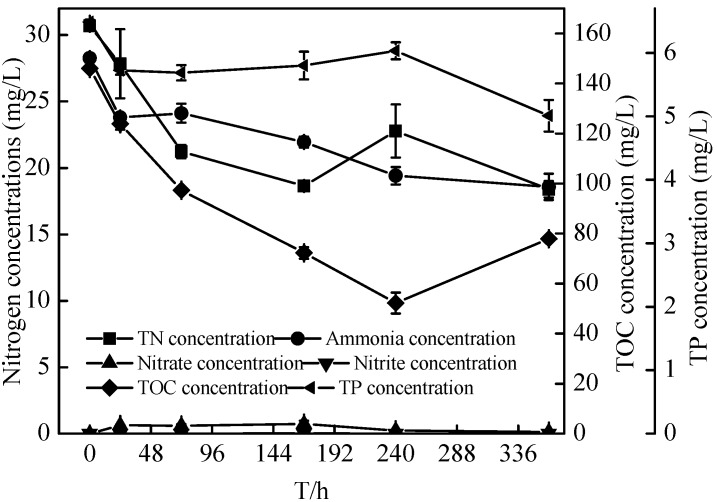
Changes in TN, ammonia, nitrate, nitrite, TP, and TOC concentrations in ammonia nitrogen medium.

In order to explore the nitrification potential of N299, strain N299 was cultured in a high ammonia-medium. Changes of various components in the flask culture are shown in Figure 10 and Figure 11. The concentration of ammonia decreased significantly, as did TN. The same trend could be seen in the removal of TOC. Simultaneously, nitrate and nitrite began to increase by nitrification, and remained as denitrification occurred without accumulation. At the end of the experiment, the ammonia decreased from 28.27 ± 0.14 to 15.79 ± 0.45 mg/L and TN decreased from 30.68 ± 0.06 to 18.40 ± 0.63 mg/L. The removal rates of ammonia and TN reached 44.12% ± 1.61% and 40.05% ± 2.04%, respectively. The TOC decreased from 146 ± 0.04 to 77.90 ± 0.31 mg/L, and nitrate and nitrite reached 0.13 ± 0.02 and 0.01 ± 0.00 mg/L. With the growth of N299, the removal rate of TP also reached 22.77% ± 3.90%. However, the C/N (TOC/TN) maintained 4.23 throughout and had a huge potential of nitrification and denitrification. Although strain N299 was not an efficient nitrifier, it demonstrated some nitrification ability without nitrate and nitrite accumulation.

### 2.6. Sterilized Reservoir Source Water Experiment of the Zoogloea sp. N299

**Figure 12:**
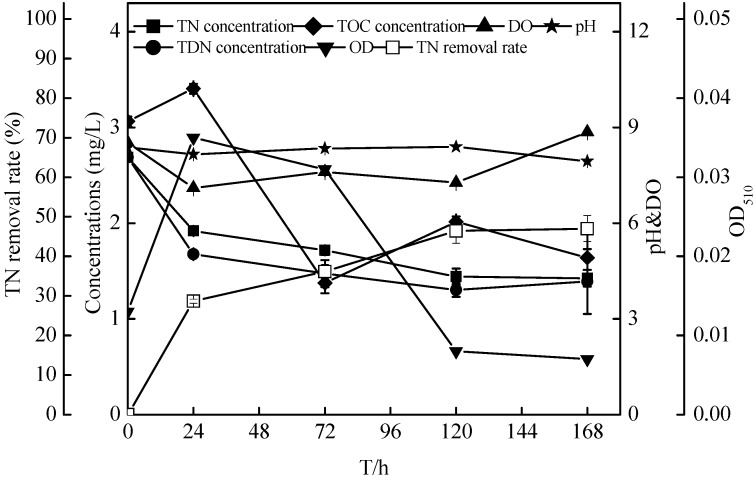
Changes in TN, TDN (total dissolved nitrogen), TOC concentrations, pH, DO (dissolved oxygen), and OD_510_ of strain N299 growth in sterilized reservoir source water.

To investigate the performance of bioremediation in micro-polluted source water, we conducted a sterilized reservoir source water experiment. The initial conditions are shown in Table 1. As shown in Figure 12, the concentration of TN decreased significantly with the growth of N299, as did TOC. The concentration of TN and TOC decreased from 2.69 ± 0.03 and 3.06 ± 0.05 mg/L to 1.43 ± 0.09 and 2.05 ± 0.05 mg/L, respectively. The same trend was seen in the removal of TDN and the decrease in DO. The TDN and DO decreased to 1.30 ± 0.07 and 7.27 ± 0.03 mg/L, respectively. The TOC/TN ranged from 1.14/1 to 1.77/1 in 24 h, 0.80/1 in 72 h, 1.40/1 in 120 h, and 1.18/1 in 168 h. The DO maintained a high level, 7–9 mg/L. under the low C/N (TOC/TN) conditions.

**Table 1:** The initial conditions of sterilized reservoir source water and non-sterilized reservoir source water experiment (values are means ± SD for triplicates, *n* = 3).

Reservoir Water	Nitrate (mg/L)		Nitrite (mg/L)		TN (mg/L)		TOC (mg/L)		pH		DO (mg/L)	
	Mean	SD	Mean	SD	Mean	SD	Mean	SD	Mean	SD	Mean	SD
sterilized reservoir source water	1.36	0.02	0.02	0.00	2.69	0.03	3.06	0.05	8.37	0.01	8.53	0.03
non-sterilized reservoir source water	1.48	0.02	0.02	0.00	2.69	0.03	3.06	0.05	8.04	0.00	4.6	0.02

### 2.7. Non-Sterilized Reservoir Source Water Experiment of the Zoogloea spp. N299

**Figure 13:**
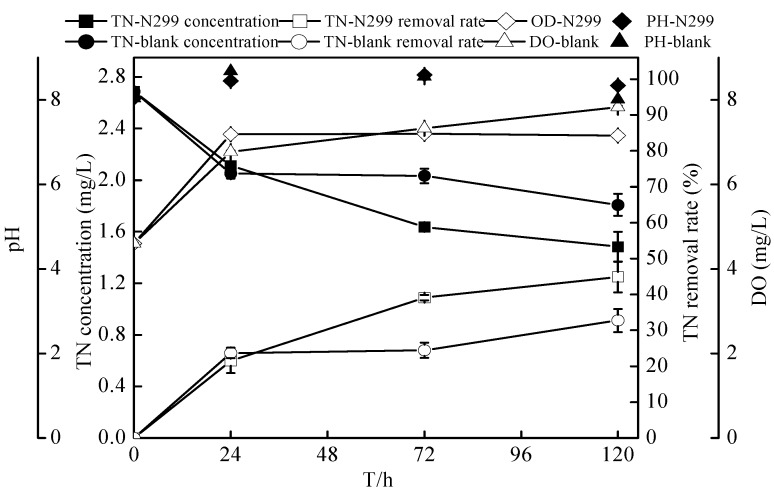
Changes of TN, pH, and DO in reservoir source water experiment. TN-N299 represents TN with *Zoogloea* sp. N299; pH-N299 represents pH with *Zoogloea* sp. N299; DO-N299 represents DO with *Zoogloea* sp. N299.

**Figure 14:**
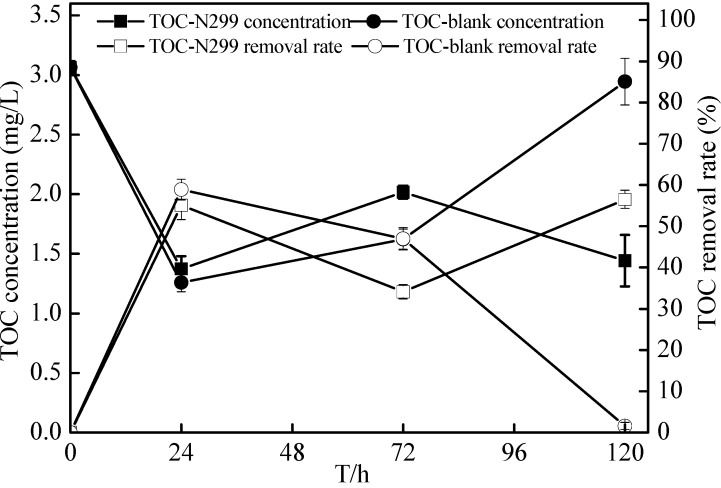
Changes of TOC concentration in reservoir source water experiment. TOC-N299 represents TOC with *Zoogloea* sp. N299.

From the sterilized reservoir source water experiment, it was shown that the N299 had a perfect adaptation to low C/N level source water and could easily survive in oligotrophic reservoir source water. This experiment studied denitrification of source water that included indigenous bacteria by N299 bacteria. The initial conditions of experiments were shown in Table 1. Changes of various component parameters in the flask culture under an aerobic condition were shown in Figure 13 and Figure 14 and Table 2. The TN of additional bacteria decreased from 2.69 ± 0.03 to 1.48 ± 0.12 mg/L in 120 h, with a removal rate of 44.88% ± 4.31%. However, the TN of a control system decreased to 1.81 ± 0.09 mg/L, and the removal rate reached 32.77% ± 3.20%. Meanwhile, in 120 h, the TOC decreased from 3.06 ± 0.05 to 1.44 ± 0.22 mg/L in the bacteria system and to 2.94 ± 0.20 mg/L in the control system. The cell optical density reached the highest level at 24 h. The DO of bacteria system was lower than the control system, while the pH was higher. During the experiment, the DO stayed at 4.6–9.0 mg/L. At the end of the experiment, the C/N ratio reached 0.97. Previous studies showed that the aerobic system expressed high denitrification ability at low DO and high C/N. From all results, the N299 was able to efficiently denitrify water at low C/N and high DO conditions, and it provided a significant opportunity for micro-polluted reservoir source water bioremediation.

**Table 2:** Changes found in a bacteria system and a control system of non-sterilized reservoir source water (values are means ± SD for triplicates, *n* = 3).

Systems/Time (h)	TN (mg/L)		TOC (mg/L)		C/N(TOC/TN) (mg/L)		pH		DO (mg/L)		OD_510_
	Mean	SD	Mean	SD	Mean	SD	Mean	SD	Mean	SD	
**Bacteria system**											
0	2.69	0.03	3.06	0.05	1.14	0.02	8.04	0.01	4.60	0.02	0.02
24	2.07	0.04	1.35	0.14	0.65	0.04	8.45	0.01	7.19	0.01	0.02
72	1.64	0.16	1.21	0.04	1.23	0.05	8.59	0.01	7.20	0.01	0.02
120	1.58	0.13	1.72	0.25	0.90	0.12	8.34	0.02	7.16	0.05	0.00
**Blank system**											
0	2.69	0.03	3.06	0.05	1.14	0.02	8.04	0.01	4.60	0.02	0.02
24	2.05	0.04	1.26	0.08	0.63	0.05	8.68	0.00	6.77	0.03	0.04
72	2.03	0.06	1.62	0.08	0.82	0.08	8.55	0.01	7.33	0.01	0.02
120	2.25	0.03	2.94	0.20	1.35	0.03	8.01	0.00	7.83	0.03	0.00

### 2.8. Effect of Different Factors on Nitrate Removal

#### 2.8.1. Carbon Sources

Carbon compounds usually serve as energy and electron sources for denitrification bacteria. Strain *Zoogloea* sp. N299 could utilize glucose, sodium succinate, sodium citrate, and sodium acetate to remove nitrate (Table 3). The nitrate removal efficiencies showed significant differences among the tested carbon sources (*p* < 0.05). Strain N299 exhibited the best nitrate removal ability (78.86% ± 0.29% in 72 h) when sodium acetate was used as the sole carbon source. Accordingly, sodium acetate was employed as the carbon source in the following experiments.

**Table 3:** Results in different factors system (values are means ± SD for triplicates, *n* = 3).

Systems	Nitrate				TN				OD_510_
	Concentration		Removal Rate		Concentration		Removal Rate		
	Mean	SD	Mean	SD	Mean	SD	Mean	SD	
**Carbon source**									
Glucose	0.99	0.01	70.79	0.24	1.01	0.02	73.58	0.40	0.046
Sodium succinate	1.57	0.01	53.55	0.23	1.83	0.02	52.10	0.40	0.036
Sodium citrate	0.74	0.01	78.32	0.18	1.67	0.03	56.43	0.65	0.049
Sodium acetate	0.72	0.01	78.86	0.29	1.08	0.02	71.76	0.54	0.063
**Temperature**									
10	1.86	0.01	45.25	0.35	2.03	0.03	47.07	0.79	0.033
20	1.09	0.01	67.75	0.36	1.19	0.03	68.99	0.65	0.046
30	0.72	0.01	78.86	0.29	1.08	0.02	71.76	0.54	0.063
**C/N ratio**									
1	2.49	0.03	17.25	0.93	3.02	0.03	21.17	0.65	0.012
3	2.15	0.01	38.43	0.29	2.97	0.03	22.56	0.78	0.02
5	1.93	0.04	43.94	1.04	2.32	0.08	39.45	2.06	0.037
8	0.72	0.01	78.86	0.29	1.08	0.02	71.76	0.54	0.063
10	0.44	0.02	85.18	0.80	0.95	0.03	75.31	0.78	0.068
**Inoculum dosage**									
2%	0.72	0.01	78.86	0.29	1.08	0.02	71.76	0.54	0.063
3%	0.77	0.01	77.23	0.41	1.16	0.02	69.68	0.54	0.055
5%	0.77	0.01	77.34	0.36	1.25	0.03	67.34	0.79	0.048
10%	0.72	0.01	78.79	0.41	1.10	0.03	71.24	0.65	0.051
**pH**									
6	1.83	0.01	45.89	0.25	2.37	0.02	38.06	0.40	0.044
7	0.72	0.01	78.86	0.29	1.08	0.02	71.76	0.54	0.063
8	0.86	0.01	74.53	0.29	1.42	0.02	62.93	0.54	0.042
9	1.97	0.01	41.82	0.29	1.88	0.03	50.97	0.65	0.043
10	2.02	0.02	40.41	0.49	2.30	0.03	39.97	0.78	0.059

#### 2.8.2. Temperature

As shown in Table 3, temperature had a pronounced effect on the nitrogen removal by strain N299 (*p* < 0.05). The nitrate removal percentage increased from 45.25% ± 0.35% at 10 °C to 78.86% ± 0.29% at 30 °C, in 72 h. Moreover, the excellent adaptability to 10–30 °C presented by strain N299 was beneficial for nitrogen removal in the natural water system.

#### 2.8.3. C/N Ratio

The influence of different C/N ratios on nitrate removal by strain N299 was shown in Table 3. Significant differences were observed among C/N ratio of 1–10 (*p* < 0.05). Maximal nitrate removal occurred at C/N ratio of 10, and reached 85.18% ± 0.80%, in 72 h. However further decrement in C/N yielded a slight decrease in nitrogen removal efficiency.

#### 2.8.4. Inoculum Dosage

The influence of different inoculums dosage (*v*/*v*) on nitrate removal by strain N299 was shown in Table 3. The nitrate removal efficiencies showed no significant differences among the tested inoculums dosage (*p* > 0.05). The nitrate removal rates were from 77.23% ± 0.41% to 78.86% ± 0.29% among inoculums dosage (*v*/*v*) of 2%–10%, in 72 h.

#### 2.8.5. pH

The pH level acted as the important parameter in simultaneous nitrification and denitrification process. In shaking cultures, neutral and slightly alkaline pH values significantly promoted nitrate removal (*p* < 0.05) (Table 3). The nitrate reached 78.86% ± 0.29% and 74.53% ± 0.29%, respectively. The optimal pH was consistent with the natural water system.

## 3. Discussion

Many studies have focused on the wastewater treatment, however, heterotrophic microorganisms often required high concentrations of organic carbon [12,42]. In contrast to domestic and industrial wastewater, source water normally contains very low contents of carbon compounds. The objective of this manuscript was to isolate aerobic denitrifiers from oligotrophic niches (oligotrophic reservoir), and evaluate the characterization of the isolate in view of its potential application in micro-polluted reservoir source water bioremediation. After 10-fold serial dilution (10^−1^, 10^−2^, 10^−3^, 10^−4^, 10^−5^, 10^−6^, 10^−7^ dilution), 0.2 mL of each bacterial suspension was blended with SM agar plate (liquid SM medium with the addition of 2% agar) and incubated at 30 °C for three days. Purified isolates were obtained via repeated streaking on fresh agar plates. To detect the denitrification performance, isolates were cultivated in SM medium with NaNO_3_ as the sole nitrogen source. Strain N299 with high nitrogen removal efficiency was obtained in this study.

According to its physiological properties and 16S rRNA gene sequences, the strain was identified as *Zoogloea* sp. N299. A phylogenetic tree was constructed based on the 16S rRNA gene sequence of strain N299 and other previously studied aerobic denitrifiers (*Klebsiella pneumoniae* strain CF-S9 [43], *Providencia rettgeri* strain YL [44], *Marinobacter* sp. H96B3, *Pseudomonas stutzeri* strain P3, *Achromobacter xylosoxidans* strain GAD3 [45], *Alcaligenes faecalis* strain NR [46], *Comamonas testosteroni* strain GAD4 [45], *Agrobacterium tumefaciens* strain LAD9 [47], and *Bacillus* sp. L7, *Rhodococcus* sp. CPZ24 [29]). Results showed that *Zoogloea* sp. N299 and *Comamonas testosteroni* strain GAD4 were in the same group, and the latter was important to efficient domestic wastewater treatment. According to the method by Guo *et al.* [31], and Kong *et al.* [48], NAP1 and NAP2 are *nap*A-specific primers [48] that correspond to the *nap*A region. The 877 bp of the *nap*A gene fragment was amplified from the strain N299 (Figure 4), which further confirmed the occurrence of aerobic denitrification by N299 and corroborated previous studies [31,48]. The specific growth of N299 is 0.22 h^−1^. Analysis with *N. europaea* (0.03–0.05 h^−1^) [49], *Pseudomonas denitrificans* (0.19–0.23 h^−1^), and *T. pantotropha* (0.28–0.45 h^−1^) under different growth conditions [15,50], and the specific growth rate of *A. faecalis* No.4 (0.2 h^−1^) [12], these results demonstrated the strong growth and substrate utilization abilities of the isolated aerobic denitrifiers.

In the pure culture medium (liquid SM, short-SM, and HNM (Heterotrophic nitrification medium) medium), the N299 strain demonstrated its strong denitrification performance and some nitrification ability without nitrate and nitrite accumulation. Using nitrate as the sole nitrogen medium, the removal rate of nitrate reached 75.53% ± 1.69% in 72 h. The removal rate of TN and TDN reached 46.79% ± 0.30% and 72.30% ± 0.52%, in 120 h. Low concentrations of nitrite (less than 6%) relative to nitrate were detected during the entire culturing period, with no nitrite accumulation. However, Xiang-Yang Xu’s study [23] showed that the removal rates of nitrate and TN reached 31.7% and 45.0% at a low substrate level (with a TOC of 48 mg/L) under the same nitrate level (4 mg/L). Growth in the basic medium, strain CPZ24 [29] removed nitrate during the rapid growth phase, and the decrease of nitrate reached 67% in 36 h. The N299 strain showed further powerful advantages. In the nitrite medium, the N299 could also carry on denitrification and remove nitrogen. The nitrite decreased from 3.76 ± 0.08 to 1.56 ± 0.01 mg/L. Aerobic denitrifiers utilized nitrite by intracellular assimilation or extracelluar reduction pathways. OD_510_ of the N299 strain (cell optical density) was increased 0.093 during the first 24 h with 1.86 mg/L nitrite and 1.78 mg/L TN consumed, respectively, suggesting that extracellular reduction had occurred, which was in agreement with co-respiration of oxygen and nitrite, as proposed by Robertson and Kuenen [15]. As the sole nitrogen and carbon source, the nitrite and TOC left 1.81 ± 0.05 and 0.24 ± 0.21 mg/L at 72 h, respectively, and the C/N (TOC/TN) decreased from 6.74/1 to 0.1/1 at 72 h; these levels were insufficient for further cell growth, and stopped all biological reactions. Few aerobic denitrifiers [51] degrade nitrite. The N299 showed its ability to utilize nitrite to grow. The ability of heterotrophic organisms to oxidize ammonium to nitrate has been linked to aerobic denitrification. Therefore, the utilization of ammonium by isolates was investigated. However, some aerobic denitrifiers could not exhibit ammonia oxidation, such as *Pseudomonas stutzeri* C3 [52]. In this study, the N299 strain could utilize ammonia as the sole nitrogen source to grow, therefore displaying heterotrophic nitrification ability. The removal rates of ammonia and TN reached 44.12% ± 1.61% and 40.05% ± 2.04%, respectively. The TN removal by the nitrification–aerobic denitrification reached 12.28 mg/L, and the ammonia reached 12.48 mg/L in ammonia medium. This indicates that the ammonia removed by N299 was distributed mainly to denitrification, not to nitrification products and assimilation products, consistent with Yang *et al.* [31], Li *et al.* [22], Chen *et al.* [29], Ni *et al.* [47], and Yu *et al.* [28]. Considering the concentration of ammonia, we would study the nitrification at a similar nitrogen concentration with the nitrate and nitrite medium in the future. Compared with other aerobic denitrification bacteria, such as, the 87% ammonium removal efficiency of all the isolated aerobic denitrifiers [30]; the ammonia removal rate of *Rhodococcus* sp. CPZ24 [29] reached 100%. Therefore, the strain N299 was not an efficient nitrifier. As with our previous aerobic denitrifier [38], which showed that the ammonia removal rate of *Rhizobium* sp. PY8 reached 58.17% in 16 days in the same medium, the N299 strain demonstrated some nitrification ability without nitrate and nitrite accumulation.

In the oligotrophic reservoir source water system. The sterilized raw water experiment showed that TN removal reached 46.99% ± 3.28%. In the non-sterilized reservoir water experiment, the TN of the bacteria system reached 44.88% ± 4.31% in 120 h. because there was a quantity of aerobic denitrifiers in the reservoir ecosystem. Therefore, the TN removal rate of the control also reached 32.77% ± 3.20%, consistent with Carter *et al.* [21]. The TN of the sterilized raw water experiment and the non-sterilized reservoir source water experiment decreased from 2.69 ± 0.03 to 1.43 ± 0.09 and 1.58 ± 0.13 mg/L in 120 h, respectively. The ideal TN concentration was below 1.00 mg/L, although, the water qualities of the experimental system did not reach Grade III based on the Chinese Surface Water Environment Quality Standard (GB3838-2002). However, compared with previous studies, such as, Liu *et al.* [31] showed that the consumptions of nitrate and ammonium were slight and TN hardly decreased at all in the added carbon source water (C/N ratio = 4); A similar study (Xu *et al.*) [23] showed the TN removal rate reached 85% in the filtered source water; Previous literature (Huang *et al.*) [36] showed removal of TN in source water reached 40% in 60 day and Wei *et al.* [39] suggested the removal rate of nitrate and TN reached 46% and 53% in 36 day. Therefore, the N299 strain demonstrated comparatively high nitrogen removal ability. Some studies showed that nitrate reductase was inhibited [27,53] in DO > 4 mg/L; most aerobic denitrifiers are able to denitrify at a DO < 3 mg/L [54,55], and few could tolerate DO at 5–6 mg/L [5,24]. However, the N299 strain could tolerate high DO at 7–8 mg/L, consistent with our previous study. From all the above results, the N299 has better tolerance to oxygen compared to other aerobic denitrifiers, could survive at low C/N and high DO, and thus shows huge application potential in the bioremediation of micro-polluted source water.

In the experiments analysing the effects of different factors on nitrate removal, acetate, due to its simple and small molecular structure, can be directly assimilated by heterotrophic microorganisms, as reported by Chen *et al.* [47], and the strain N299 had the best nitrate removal performance when sodium acetate was employed as the carbon source. Zaitsev *et al.* [56] showed that nitrification and denitrification rates doubled with every 10 and 4 °C increase, respectively. Nitrate removal rates did not significantly increase if the temperature increased from 20 to 30 °C. In natural water environments, the temperature was rarely higher than 30 °C, therefore 20 °C was used for the further research. Most investigations on nitrogen removal by heterotrophic nitrifying-denitrifying bacteria were conducted at C/N ratio of 10 [12,42]. It was suggested that insufficient carbon supply impairs both microbial growth and electron donors for denitrification [57,58]. Hence, fewer requirements for C/N ratio in the reservoir source water experiment would be favorable for the nitrogen removal of oligotrophic source water. The nitrate removal efficiencies showed no significant differences among the tested inoculums dosage (*p* > 0.05), which demonstrated a positive influence on the practical application. The nitrate removal rate reached 78.86% ± 0.29% at pH 7 and 74.53% ± 0.29% at pH 8, not significantly, which was consistent with *Pseudomonas stutzeri* strain T1 [31]. Namely, neutral or slightly alkaline environments were beneficial for nitrogen removal. These pH values were comparable with that of Zhoucun reservoir, which was approximately neutral or slightly alkaline.

## 4. Experimental Section

### 4.1. Samples

Reservoir sediment samples were collected from the Zhoucun drinking water reservoir (34°56'38.74''N, 117°41'14.13''E). In June 2011, surface sediments were collected at a deep layer of 0 to 10 cm using a sterilized Petersen stainless steel grab sampler [38,40]. The reservoir source water was sampled. The samples were stored in black plastic bags at 4 °C, and transferred to the Key Laboratory of Northwest Water Resource, Environment and Ecology, Xi’an University of Architecture and Technology.

### 4.2. Enrichment Cultures and Isolation of Aerobic Denitrifiers

The 100 mL sludge sample was added into 700 mL heterotrophic enrichment denitrification broth medium (HEDM) at pH 7.0–7.5: CH_3_COONa (0.5 g/L); NaNO_3_ (0.1 g/L); K_2_HPO_4_·3H_2_O (0.1 g/L); CaCl_2_ (0.05 g/L); MgCl_2_·6H_2_O (0.05 g/L) [38,40]. Every three days we removed the liquid medium, reduced the concentration of the medium by one-tenth, and put the new medium into the sludge sample, until the concentration of the HEDM became one-tenth the first concentration. The enrichment of aerobic denitrifiers lasted almost one month [59]. The temperature and DO of the enrichment cultures were controlled at room temperature and nearly 5 mg/L. The enrichment sludge suspension was sampled via gradient dilution, and the gradient dilutions were carried on as follows: 10^−1^ dilution (1 mL enrichment sludge suspension added to 9 mL sterile distilled water); 10^−2^ dilution (1 mL 10^−1^ dilution suspension added to 9 mL sterile distilled water); 10^−3^ dilution (1 mL 10^−2^ dilution suspension added to 9 mL sterile distilled water); 10^−4^ dilution (1 mL 10^−3^ dilution suspension added to 9 mL sterile distilled water); 10^−5^ dilution (1 mL 10^−4^ dilution suspension added to 9 mL sterile distilled water); 10^−6^ dilution (1 mL 10^−5^ dilution suspension added to 9 mL sterile distilled water); 10^−7^ dilution (1 mL 10^−6^ dilution suspension added to 9 mL sterile distilled water). The resultant bacterial suspension was streaked on a screening medium plates and incubation temperature of 30 °C for 3 days. A screening medium (SM) [36] plate at agar (20 g/L); pH 7.0–7.5; CH_3_COONa (0.1 g/L); NaNO_3_ (0.02 g/L); K_2_HPO_4_·3H_2_O (0.02 g/L); CaCl_2_ (0.01 g/L); MgCl_2_·6H_2_O (0.01 g/L). Separate colonies were picked and purified by repeated streaking on fresh agar plates. The isolates were harvested and cultivated in SM medium with NaNO_3_ as the sole nitrogen source in order to detect the aerobic denitrifying bacteria performance. An isolate N299 with high nitrogen removal efficiency was obtained in this study, and SM slant medium at 4 °C and on SM Glycerin medium at −20 °C.

### 4.3. Analysis of 16S rRNA Gene Sequence

The 16S rRNA sequence of *Zoogloea* sp. N299 was obtained via PCR. The PCR used the primers [60]: 7F 5'-CAGAGTTGATCCTGGCT-3', and 1540R 5'-AGGAGGTGATCCAGCCGCA-3'. The PCR reaction mix consisted of the following reagents, which were extracted and sequenced by Sangon Biotech (Shanghai) Co., Ltd. (Shanghai, China): 5× Buffer (with Mg^2+^) (2.5 μL), DNA template (0.5 μL), dNTP (each 2.5 mM), Taq DNA Polymerase (0.2 μL), and sterile nuclease free water to 25 μL. The PCR was carried out as follows: 94 °C for 4 min for one cycle and then 30 cycles of denaturation at 94 °C for 45 s, annealing at 55 °C for 15 s, and extension at 72 °C for 1 min. After a final extension at 72 °C for 10 min, reactions were stored at 4 °C. The Seqman was used to align the sequences. Homology searching of the sequences in GenBank was performed using BLAST (http://blast.ncbi.nlm.nih.gov/Blast.cgi). A neighbor-joining tree was constructed in MEGA5.0 program using neighbor-joining (NJ) method with the maximum composite likelihood model and 1000 bootstrap replicates [35]. Culture strains highly similar to the genera are listed in Figure 2. Finally, The sequence of the N299 strain has been submitted into GenBank for its accession number; the strain was also deposited to the China General Microbiological Culture Collection Center (CGMCC).

### 4.4. Amplification of the napA Gene

The *nap*A was amplified with the forward primers NAP1: 5'-TCTGGACCATGGGCTTCAACCA-3' [48], and NAP2: 5'-ACGACGACCGGCCAGCGCAG-3' [48]. The PCR reaction mix (50 μL) consisted of 2× Taq Mastermix (0.1 U Taq Polymerase/μL), 500 μM dNTP, 20 mM Tris-HCl (pH 8.3), 100 mM KCl, 3 mM MgCl_2_ (25 μL), NAP1 (1 μL), NAP2 (1 μL), and sterile nuclease-free water to 50 μL. Conditions used for this reaction were as per [48]: initial start at 94 °C for five min for one cycle, followed by 30 cycles of denaturation at 94 °C for 30 s, annealing at 59 °C for 30 s, and extension at 72 °C for 1 min. After a final extension at 72 °C for 7 min, reactions were stored at 4 °C. PCR products were separated using 1.0% agarose gel electrophoresis and then stained with ethidium bromide for visualization.

### 4.5. Growth Characteristics of the Zoogloea sp. N299

The growth characteristics of the isolated strain N299 were determined through measuring OD_510_ in a shake flask experiment in which 400 mL of the liquid SM medium was put in 1000 mL shake flasks, inoculated with 4 mL of strain pre-culture, and then cultivated at 30 °C. During incubation, 3 mL culture was removed periodically for the determination of cell optical density. The aerobic denitrifying bacteria N299 was pre-cultured for 24 h in 50 mL liquid SM medium (without agar) in a 100 mL Erlenmeyer flask at 30 °C and 120 rpm in order to be activated [36]. According to the study conducted by Duu-Jong Lee [51], the logistic growth equation describes the cell growth curve:

y(*t*) = *a*/[1 + (*a*/*c* − 1)exp(−μt)] (1)

where *t* is time (h); y(*t*) is bacterial cell density at *t* h (OD); μ is the maximum specific cell growth rate (h^−1^); and *a* is the maximum bacterial cell density (OD); *c* is the bacterial cell density (*t* = 0). Correlation analysis using OriginPro (Ver. 8.0, OriginLab Corporation, Northampton, MA, USA) yielded.

### 4.6. Nitrogen Removal Performance in Pure Culture Medium System

The pre-cultured N299 strain was inoculated in 10% (*v*/*v*) into 150 mL liquid SM, short-SM, and HNM of 250 mL Erlenmeyer flask at 30 °C, 120 rpm, respectively. The nitrate, nitrite, TN, TDN, TP, TOC concentrations, and cell optical density (OD) were measured to reflect the denitrification performance of the N299 strain. All parameters were measured in triplicate (*n* = 3). The SM medium included, at pH 7.0–7.5: CH_3_COONa (0.1 g/L), NaNO_3_ (0.02 g/L), K_2_HPO_4_·3H_2_O (0.02 g/L), CaCl_2_(0.01 g/L), and MgCl_2_·6H_2_O (0.01 g/L). A short SM medium [59], at pH 7.0–7.5: CH_3_COONa (0.1 g/L), NaNO_2_ (0.018 g/L), K_2_HPO_4_·3H_2_O (0.02 g/L), CaCl_2_ (0.01 g/L), MgCl_2_·6H_2_O (0.01 g/L). Heterotrophic nitrification medium (HNM) [38] was also prepared at pH 7.0–7.5: CH_3_COONa (0.5 g/L), NH_4_Cl_4_ (0.1 g/L), K_2_HPO_4_·3H_2_O (0.1 g/L), CaCl_2_ (0.05 g/L), and MgCl_2_·6H_2_O (0.05 g/L).

### 4.7. Nitrogen Removal Performance in Oligotrophic Reservoir Source Water System

In order to investigate whether adding agents could purify the sterilized oligotrophic reservoir source water and to study whether bacteria in source water affected the N299’s denitrification, sterilized reservoir source water and non-sterilized source water experiments were carried out. The pre-cultured N299 was inoculated in 10% (*v*/*v*) into 150 mL sterilized oligotrophic reservoir source water and non-sterilized oligotrophic source water of 250 mL Erlenmeyer flask at 30 °C at 120 rpm. The TN, TDN, TOC, cell optical density, pH, and DO were measured to reflect the denitrification performance of N299. All parameters were measured in triplicate (*n* = 3).

### 4.8. Effect of Different Factors on Nitrate Removal

The heterotrophic aerobic denitrification characteristics of isolated strain were determined under different culturing conditions, including carbon source, Temperature, C/N, inoculums dosage (*v*/*v*) and pH. Glucose, sodium succinate, sodium citrate, and sodium acetate were used to explore the effects of carbon source on nitrate removal. To observe the effect of temperature on nitrate removal, the experiment was performed within the range of 10–30 °C. The effect of C/N (sodium acetate as carbon source) on nitrate removal was examined by adjusting the ratio between 1 and 10 with a fixed amount of 3.54 mg/L NO_3_^−^-N. The influence of inoculums dosage (*v*/*v*) on nitrate removal was conducted by changing the inoculums dosage to 2%, 3%, 5% and 10%. The effect of pH on nitrate removal was examined in 6, 7, 8, 9 and 10. All parameters were measured in triplicate (*n* = 3).

### 4.9. Analytical Methods

The optical density of the culture broth was measured at 510 nm (OD_510_) using a spectrophotometer (DR6000, HACH Company, Loveland, CO, USA) [61]. Nitrite was determined by *N*-(1-naphthalene)-diaminoethane photometry method [62]. TN and nitrate were measured by the hydrochloric acid photometry method [62]. TP was measured by ammonium molybdate spectrophotometric method [63]. TOC determined by TOC analyzer (ET1020A, Shanghai, China). SEM analyzed by S-3400N (Hitachi, Tokyo, Japan). The samples of nitrate, nitrite, TDN, TOC and TP were filtered using a 0.45 μm cellulose-acetate filter for removing bacteria. pH was measured by HQ11d (HACH Company) and DO was measured by HQ30d (HACH Company). Surface sediments were collected at a deep layer of 0 to 10 cm using a sterilized Petersen stainless steel grab sampler [38,40]. Phylogenetic analysis was constructed in MEGA5.0 program using a neighbor-joining (NJ) method and the maximum composite likelihood model [38].

### 4.10. Statistical Analyses

Data are presented as means ± SD (standard deviation of means), and analyzed by one-way ANOVA with Tukey’s HSD test (*p* < 0.05) using SPSS software (Ver. 20.0, IBM Corporation, Armonk, NY, USA).

## 5. Conclusions

The newly isolated indigenous aerobic denitrifier, N299 strain was named as *Zoogloea* sp. N299 through the 16S rRNA. The 877 bp of the *nap*A gene fragment was amplified from the strain N299, and the specific growth rate was 0.22 h^−1^. The N299 strain showed its ability to utilize nitrate and nitrite as sole nitrogen source to grow. The N299 strain can also utilize ammonia as a sole nitrogen source to grow, and has heterotrophic nitrification ability. Under the low C/N (1.14/1) and high DO (7–8 mg/L) conditions, the N299 showed an efficient denitrification performance and strong adaptability to tolerate low C/N and high DO. The strong adaptability of strain N299 to neutral or slightly alkaline pH and low temperature conditions make it a promising candidate for treating micro-polluted source water in natural regions. It could utilize the organics of water to remove nitrogen to survive. This study thus provides significant information for bioremediation of micro-polluted reservoir source water.
